# Neural Networks Track the Logical Complexity of Boolean Concepts

**DOI:** 10.1162/opmi_a_00059

**Published:** 2022-09-01

**Authors:** Fausto Carcassi, Jakub Szymanik

**Affiliations:** Seminar für Sprachwissenschaft, Tübingen, Germany; Institute for Logic, Language, and Computation, Universiteit van Amsterdam, Amsterdam, Netherlands

**Keywords:** artificial neural networks, Boolean complexity, category acquisition

## Abstract

The language of thought hypothesis and connectionism provide two main accounts of category acquisition in the cognitive sciences. However, it is unclear to what extent their predictions agree. In this article, we tackle this problem by comparing the two accounts with respect to a common set of predictions about the effort required to acquire categories. We find that the two accounts produce similar predictions in the domain of Boolean categorization, however, with substantial variation depending on the operators in the language of thought.

## INTRODUCTION

Category acquisition is one of the most important topics in cognitive science. Various accounts of category acquisition have been proposed, among which are the language of thought hypothesis (LOTH) and connectionism. The LOTH claims that human thinking consists of manipulation of symbols in a language (Fodor, 1975). Connectionism is a program attempting to explain various aspects of cognition using artificial neural networks (ANNs) (see Sutton, 1995, for a discussion of levels of description and explanation in connectionism). The relations between these two accounts have been the subject of extensive philosophical debate, but there is little empirical data directly comparing the two approaches.^1^

In this article, we explore the relation between these two accounts of cognition with respect to categorization. In particular, the two accounts can be compared with respect to their empirical predictions, leading to the following question:

**Question.** *Do connectionism and the *LOTH* have similar empirical import in the domain of category acquisition?*

Category acquisition is a complex phenomenon with various empirically interesting features. The first step in answering this question is then finding an aspect of category acquisition for which both accounts make predictions.

Connectionism makes predictions for various aspects of category acquisition. Among others, ANNs have been used to predict and explain base-rate neglect (Estes et al., 1989; Gluck & Bower, 1988), stages of learning (Rumelhart & McClelland, 1987), and, in more recent work patterns, in categorization of individuals with autism spectrum disorder (Dovgopoly & Mercado, 2013). Recent work on *deep* learning, involving ANNs with many layers, shows that there are interesting connections between cognitive processing and the representations formed at various depth in ANNs (Guest & Love, 2019). Most importantly for the present purposes, connectionism predicts a correlation between the effort required to learn a category by ANNs and by humans (Bartos, 2002; Kruschke, 1991).

The LOTH also makes empirical predictions about various aspects of category acquisition. For example, it predicts which inductive generalizations will be made at each stage of learning (Kemp et al., 2008). Much of the previous literature has focused on predictions concerning the effort required to learn each category. Specifically, categories encoded by longer formulas in the language of thought (LOT) will take longer to learn. Previous work has verified this experimentally by comparing the learning efforts predicted by the model with those in human participants. A foundational paper in this approach is Feldman (2000), which considers the minimal formulas required to encode a set of Boolean functions in a specific LOT.^2^ Goodman et al. (2008) develops similar ideas in a Bayesian model. While both papers are relevant to the model we present here, they only consider one specific shape for the formulas (namely, disjunctive normal form), rather than exploring the possible LOTs.^3^

In sum, given some natural assumptions we discuss below, both LOTH and connectionism make predictions about the *effort* required to acquire categories, which offers a natural set of empirical predictions to consider. In order to answer the question above, we therefore compare for each category in the conceptual domain the learning efforts predicted by the two accounts. We find a much better fit between the learning effort of simple ANNs and Boolean logical complexity than would be expected by chance. At a minimum, this result constitutes evidence that these accounts are compatible with each other. Moreover, if alternative combinations of accounts show worse fit than the one we observed, our results constitute evidence that connectionism and the language of thought hypothesis are particularly compatible: evidence in favor of one will also make the other more plausible.

## MODELING CATEGORY ACQUISITION

Our aim is to compare connectionism and LOTH in the domain of category acquisition. We do this by finding the predictions of the two accounts separately via computational models and then comparing them. First, we discuss the model of categories, present a model of category acquisition in the two accounts, and derive the behavioral predictions with respect to which the accounts can be compared. Then, we present our method for comparing the predictions made by the two accounts.

Categorization in our model relies on identifying which properties define a category and in which combination. Therefore, we start with a set of binary properties, for example, “small” or “square.” An object can be thought of as a set of properties.^4^ For instance, an object might be identified by being small and square. Two objects are then only distinguishable via a difference in which properties they have. Therefore, given *n* properties 2^*n*^ possible objects can be constructed. Finally, we define a category as a set of objects, namely, those objects that belong to the category. Therefore, there will be one category for each possible set of objects. With *n* properties, there are 2^(2^*n*^)^ possible categories. In the models below, we will include four properties, which can construct 16 possible objects and 65,536 possible categories.^5^

**Figure 1.**
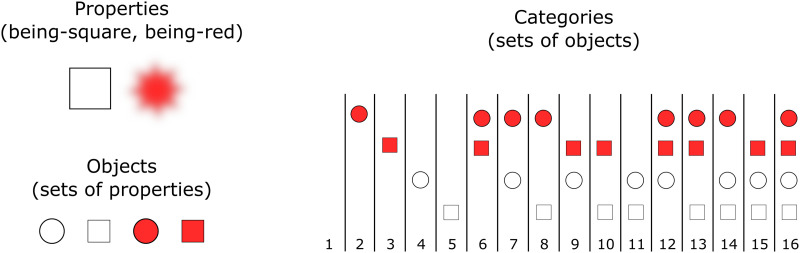
**Example of the setup with two properties: square and red (top left).** For simplicity, we represent nonsquareness as circle and nonredness as white. An object is defined as a set of properties or their negation (bottom left). Since we have two properties, four possible objects can be distinguished. A category is then defined as a set of objects (right). With four objects, 16 categories can be defined. For instance, category 1 is the empty category, containing no objects. Category 7 contains any object that is a circle, regardless of its being red. Category 12 contains all objects that are either red or a circle.

Figure 1 gives an example of the setup for two properties. The example shows two properties *p* (being-square) and *q* (being-red). Four objects can be defined with these two properties: {*p*, *q*}, {*p*, ¬*q*}, {¬*p*, *q*}, and {¬*p*, ¬*q*}. Any set of these four objects is a category, and there are 16 possible categories. For instance, one category does not contain any of the objects (category 1 in Figure 1), and one category contains only the red objects (category 6).

### Boolean Fragment of the Language of Thought

The case of Boolean conceptual domain has received particular attention starting from Boole (1854), because of its tractability and importance in thinking. The LOTH offers a natural approach to modeling categorization in the Boolean conceptual domain. In practice, our aim is to construct an LOT whose expressions encode Boolean functions: functions from truth values (specifying for each property whether the object has it) to a truth value (specifying whether the object belongs to the category). Each expression in the LOT then encodes a category, namely, the set of those objects that verify the corresponding Boolean function.^6^ To see how this works in practice, consider the example of the Boolean LOT with the two properties pictured in Figure 1 and the operators ¬, ∧, and ∨. Category 13 ({{¬*p*, *q*}, {*p*, *q*}, {*p*, ¬*q*}}) can, for example, be expressed in this LOT simply as *p* ∨ *q*.

Previous work has analysed the complexity of Boolean formulas in specific LOTs. For instance, Feldman (2003a) categorizes Boolean functions up to four inputs in an LOT with negation, conjunction, and disjunction, as well as their formulation in disjunctive normal form (DNF). Feldman (2003b) shows that in humans Boolean complexity, calculated with a specific LOT, correlates with learning effort. Feldman (2006) deepens these results by defining a different coding pattern for these formulas. However, previous literature has not systematically explored the set of possible LOTs.

Since the same category is encoded by formulas with possibly different lengths in LOTs with different operators, and therefore different LOTs make different empirical predictions for category acquisition, we must define the space of possible LOTs to be considered in the model below. Different LOTs are distinguished in our model by different syntactic primitives. The primitives include literals referring to the properties and the Boolean operators to encode functions of the properties. In the following we assume a set of four properties, which are identical across LOTs, and we let the operators vary across LOTs. To reduce the space of LOTs to a manageable size, we only consider binary and unary operators.

In order to produce behavioral predictions about category learning from this picture, we rely on the natural assumption that categories that are expressed by more complex formulas in the LOT are harder to learn. Therefore, while each category can be expressed by multiple (and possibly infinitely many) formulas in a given LOT, here we will assume that the relevant formula is the *shortest* among the formulas that encode the category, following among others (Feldman, 2000). This is also in line with previous work emphasizing the importance of complexity in cognition (Chater & Vitányi, 2003; Grünwald, 2007). This assumption implies that LOTs that produce the same minimal formula length across all categories are equivalent for our purposes.^7^

Given these restrictions, we can immediately exclude some operators, on three grounds. First, some operators would not appear in any minimal formula even in LOTs that contain them, because any formula that contains them is equivalent to a shorter formula without them. These operators are (1) affirmation, the unary operator that simply return the truth value of its argument and can therefore be substituted by the argument alone, (2) left-affirmation, the binary operator that returns the truth value of its left argument and can therefore be substituted by the left argument alone, and (3) right-affirmation, which is similar to left-affirmation for right argument. Second, some operators produce the same minimal formula lengths as an LOT with just negation instead. These operators are (1) the binary left-negation, which can be substituted by the negation of the left argument and (2) the binary right-negation, which can be substituted by the negation of the right argument. Third, any LOT with ← produces the same minimal formula lengths as an LOT that is the same up to substitution of ← for →, and similarly for ↚ and ↛. Therefore, we exclude ← and ↚ and only keep → and ↛ (see Table 1 for the meaning of the symbols).

**Table 1.** The Nine Operators That Appear in at Least One Minimal Formula in Some Boolean LOTs

Name	Symbol
conjunction (and)	∧
disjunction (or)	∨
conditional	→
negated conditional	↛
biconditional	↔
negated biconditional	↮
negated conjunction	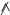
negated disjunction	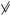
negation	¬

As mentioned above, we assume that all LOTs can express the same set of properties. While this modeling choice might seem arbitrary, it is in fact required for our model. LOTs expressing different sets of properties would be incapable of describing the same sets of objects and categories, and therefore the complexity of the corresponding expressions could not be compared with each other.

These restrictions leave us with nine out of the 16 possible operators, listed in Table 1. The set of LOTs is then the powerset of the set of these nine operators, that is, any set constructed with zero or more of these nine operators. In principle, 2^9^ = 512 LOTs can be constructed from these nine operators. We reduce the number of LOTs further by assuming that the LOTs are *Boolean bases*, that is, that every category can be expressed with them.^8^ This leaves 468 LOTs. Finally, we exclude LOTs that in practice produce the same minimal formula length for all categories, leaving a total of 358 LOTs with unique complexity profiles.

We assume that the learning effort predicted by the LOTH given a specific LOT is a monotonically increasing function of these minimal formula lengths in that LOT (see Table 2 for some examples of minimal formulas). Since we do not know which function this is, we only interpret the length-ranks of these minimal formulas as the predicted complexity ranks: the categories with the shortest minimal formulas will be easiest, the categories with the second shortest minimal formulas will be the second easiest, and so on. Therefore, we take the LOTH with a specific LOT to predict the rank of acquisition complexity.

**Table 2.** Minimal Formulas for All Categories Definable With Two Properties (p and q), for Three Language of Thoughts (LOTs): A Minimal LOT Containing Only the Operator “nand,” an English-like LOT with “not,” “and,” “or,” and “nor,” and an LOT With a Parity Operator (Negated Biconditional)*

Categories	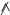	¬, ∧, ∨, 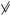	¬, ∨, ↮
1100	*p*	*p*	*p*
1010	*q*	*q*	*q*
0011	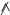 (*p*, *p*)	¬(*p*)	¬(*p*)
0111	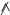 (*p*, *q*)	¬(∧(*p*, *q*))	∨(¬(*p*), ¬(*q*))
0101	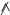 (*q*, *q*)	¬(*q*)	¬(*q*)
1111	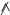 (*p*, 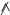 (*p*, *p*))	∨(*p*, ¬(*p*))	¬(↮ (*p*, *p*))
1011	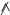 (*p*, 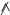 (*p*, *q*))	∨(*q*, ¬(*p*))	∨(*q*, ¬(*p*))
1101	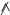 (*q*, 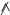 (*p*, *p*))	∨(*p*, ¬(*q*))	∨(*p*, ¬(*q*))
1110	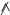 ( 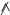 (*p*, *p*), 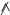 (*q*, *q*))	∨(*p*, *q*)	∨(*p*, *q*)
1000	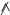 ( 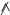 (*p*, *q*), 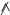 (*p*, *q*))	∧(*p*, *q*)	¬(∨(¬(*p*), ¬(*q*)))
1001	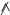 ( 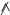 (*p*, *q*), 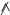 ( 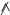 (*p*, *p*), 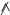 (*q*, *q*)))	∨(∧(*p*, *q*), 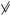 (*p*, *q*))	¬(↮ (*p*, *q*))
0000	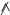 ( 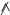 (*p*, 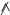 (*p*, *p*)), 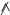 (*p*, 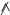 (*p*, *p*)))	∧(*p*, ¬(*p*))	↮ (*p*, *p*)
0100	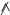 ( 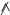 (*p*, 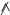 (*p*, *p*)), 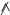 (*p*, 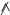 (*p*, *q*)))	∧(*p*, ¬(*q*))	¬(∨(*q*, ¬(*p*)))
0010	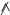 ( 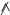 (*p*, 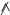 (*p*, *p*)), 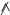 (*q*, 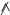 (*p*, *p*)))	∧(*q*, ¬(*p*))	¬(∨(*p*, ¬(*q*)))
0110	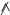 ( 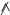 (*p*, 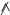 (*p*, *q*)), 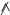 (*q*, 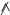 (*p*, *p*)))	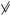 (∧(*p*, *q*), 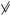 (*p*, *q*))	↮ (*p*, *q*)
0001	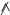 ( 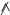 (*p*, 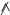 (*p*, *p*)), 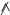 ( 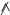 (*p*, *p*), 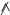 (*q*, *q*)))	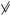 (*p*, *q*)	¬(∨(*p*, *q*))

* Each row corresponds to one possible category, conveyed by the first column as a Boolean vector over the truth table for p and q (in left-to-right order: p and q, p and not q, not q and p, not q and not p). It is instructive to compare the behavior of the three LOTs for the different categories. First, since all LOTs have lexical means to refer to the properties directly, the two categories that refer to all and only the objects having one of the properties have the same minimal formula in all languages. Second, the longest formulas for an LOT with fewer operators (first columns) are longer than for an LOT with more operators (second columns). Third, some categories which are among the simplest in one LOT [e.g., “and(p, q)”] can be among the most complex for another [e.g., “not(or(not(p), not(q)))”].

### Connectionism

Category acquisition has been a historically crucial case in the study of ANNs, specifically in the case of the *parity function*, expressed in the binary case by the negated biconditional ↮.^9^ One n-bits parity function *f* : $\overset{n}{\underset{i=1}{\times }}$ {0, 1} → {0, 1} is defined as:

$$f\left(x_{1},\ldots ,x_{n}\right)=\left\{\begin{array}{ll} 1 & \text{if}\;\sum_{i=1}^{n}x_{i}\;\text{is}\;\text{even}.\\ 0 & \text{else}. \end{array}\right.$$

and the other parity function is its negation *f*′(*x*_1_, …, *x*_*n*_) = 1 − *f*(*x*_1_, …, *x*_*n*_). The *parity problem* in the context of ANNs is the problem of constructing an ANN that implements a parity function. An early version of ANNs, the *Perceptron* (White & Rosenblatt, 1963), was shown by Minsky and Papert (1972) incapable of solving the parity problem. Later, Rumelhart et al. (1986) showed that backpropagation and nonlinear activation functions allow an ANN to solve the parity problem.^10^

More recent work has produced more insights into Boolean learning by ANNs.^11^ Sprinkhuizen-Kuyper and Boers (1996) shows that some simple ANN architecture, across all possible inputs, encode the trivial functions (always output 0 or always output 1) very often and the parity functions very rarely. Mingard et al. (2020) also study the inductive biases of simple ANN, by studying the distribution of Boolean functions obtained with randomly initialized weights. They find a bias for Boolean functions with low entropy, that is, functions that are verified by few or many inputs. Moreover, they find that given some specific entropy levels, ANNs have a prior bias for Boolean functions with low Lempel-Ziv complexity.

Rather than focusing on the distribution of Boolean functions for random initializations, we look at the effort required to acquire different categories. Therefore, we train ANNs on each possible Boolean function (A slight complication arises with respect to the interpretation of the input and output of the ANNs, which we describe in the Appendix.). Each network has four input neurons, one for each property, and one output neuron, which encodes the network’s confidence that the object described by the input belongs to the category. Each network has two hidden layers of 16 neurons each and ReLU (rectified linear unit) activation functions, except on the last layer, where a sigmoid function is applied to squeeze the output in the (0, 1) interval. We used binary cross entropy to measure the difference between the network’s output and the true output as determined by the category. To train the network, we use the Adam optimizer, a popular algorithm for gradient-descent (Kingma & Ba, 2015). We made these design choices so that the networks had enough expressive power to represent every category. Future work will analyze the effect of architecture choices on the results presented below.

For each of these categories, we train 25 ANNs. Each training has batch sizes of eight and 400 epochs. We measure the effort needed by an ANN to learn a specific category by taking the average loss across epochs and batches.^12^ Therefore, for each category we have 25 samples of our measure of complexity of ANN learning effort for the category.

## COMPARING THE TWO ACCOUNTS’ PREDICTIONS

**Figure 2.**
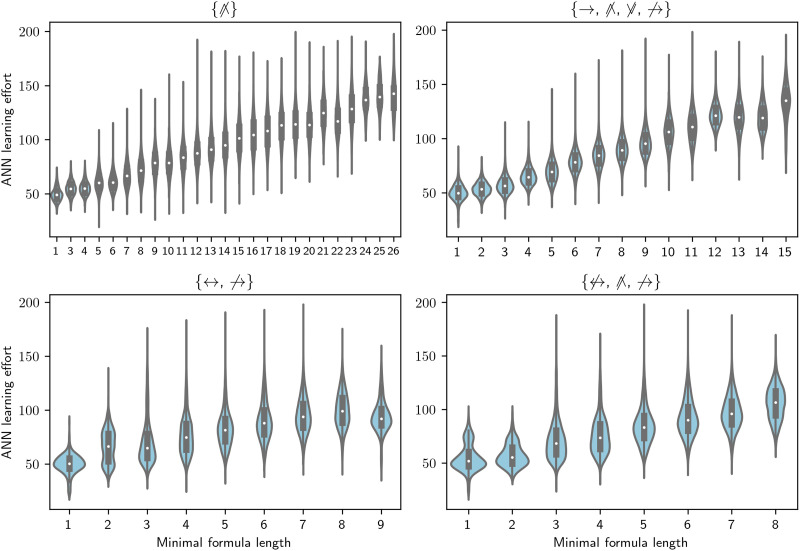
**Artificial neural network (ANN) learning effort as a function of minimal formula length for all categories, for four language of thoughts (LOTs).** The title of each subplot indicates the operators in the displayed LOT. For instance, the LOT displayed in top left plot only contains negated conjunction, while the bottom right LOT contains negated biconditional, negated conjunction, and negated conditional. The plot displays some differences in the way different LOTs encode formulas. First, LOTs with more operators generally have a smaller range of minimal formula lengths, since even the most complex categories for that LOT can be described with shorter formulas. For instance, the length of minimal formulas for the LOT with just negated conjunction (top left) go up to 26, while for an LOT with four operators they only go up to 15 (top right). Second, while all LOTs show a positive rank correlation between the two variables, there is considerable variation in the strength of this correlation. Namely, LOTs with the parity operators (bottom subplots) have a weaker rank correlation than LOTs without them.

In the previous section, we have discussed how to model category acquisition within a connectionist account and an LOT account. Moreover, we have extracted for both of these accounts empirical predictions of the effort of acquiring each category. Recall that in our simulations we considered all categories definable with the set of objects that can be constructed with four features or, equivalently, the set of possible functions *f* : Bool^4^ → Bool. Figure 2 shows examples of the relation between the length of the minimal formulas in four LOTs and the ANN learning efforts for the corresponding categories. Since the ANN learning efforts are the same for all LOTs, each plot shows a reordering of the learning efforts that depends on the minimal formula lengths in the LOT. While all four displayed LOTs show an increasing trend, there are also substantial differences between them. This is because different sets of operators produce different formulas (and therefore different formula lengths) for different categories, so that the same category might be expressible compactly in one LOT but require a long formula for another LOT. This raises the question of whether there is across LOTs an overall correlation between minimal formula length and ANN learning effort.

We hypothesize an overall monotonically increasing relation between ANN learning effort and logical complexity. Since this hypothesis does not imply or assume a functional relation between the two, a parametric measure such as Pearson’s correlation coefficient *r* would be inappropriate. Rather, we use a nonparametric measure, that is, Spearman’s rank correlation coefficient *ρ*, which is the Pearson’s correlation between the *ranks* of the two variables. The *ρ* quantifies how well the relation between the two variables can be encoded by a monotonic function. When *ρ* = 1, the two samples can be perfectly described by a monotonically increasing function, while when *ρ* = −1, a monotonically decreasing function. Our hypothesis can then be operationalized as the claim that there is a positive rank correlation between the ANN learning effort and the logical complexity of categories.

A welcome side effect of using rank correlations is that the results, since they depend only on complexity ranks, are not sensitive to any monotonic transformation of the involved variables. Therefore, while the particular measures of learning effort we chose (average loss across epochs and batches, see footnote 12 for discussion) is not uniquely identified by theoretical considerations, our results are not sensitive to other choices, as long as they preserve the complexity ranks. Therefore, the results are robust to different accounts of how simplicity affects learning—for example, Bayesian learning (Piantadosi et al., 2016) or minimum description length (Grünwald, 2007)—as long as simpler categories are easier to learn.

**Figure 3.**
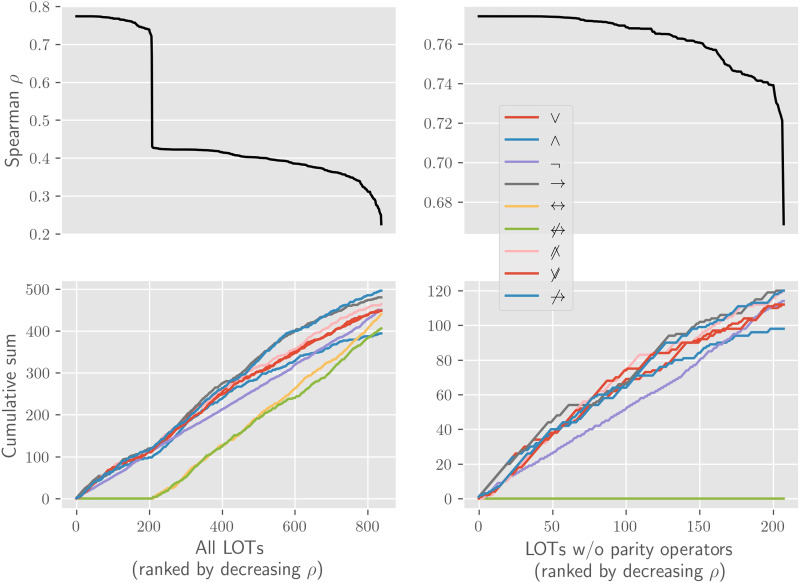
**The model’s aim is to measure the extent to which artificial neural network (ANN) learning effort and logical complexity (given a language of thought [LOT]) agree across categories.** In order to quantify the level of agreement between them, we use the rank correlation *ρ*, which is the linear correlation between the category-wise complexity ranks in the two measures, and is robust to monotonic transformation of the complexity measures. The top left plot shows *ρ* between learning efforts and minimal formula length for all LOTs, where LOTs are ordered by decreasing rank correlation (as can be seen by the strictly decreasing value of *ρ*). Two things ought to be noticed about this subplot. First, *ρ* is always positive: across all LOTs there is a positive rank correlation between logical complexity and ANN learning effort. Second, around 200 there is a stark decrease in *ρ*. The reason for this stark decrease can be seen in the bottom left subplot, which shows the cumulative number of occurrences of each of the nine operators from Table 1 (i.e., the total number of time each operator appeared in the LOTs with at least that level of *ρ*). For instance, the line for *A* when *x* = 3 shows the number of times *A* occurs in the three LOTs with the highest correlation to ANN learning efforts. The bottom left plot then shows that the introduction of parity operators causes the stark reduction in correlation: the stark decrease can be seen to correspond to the appearance of parity operators. The right plots restrict the corresponding left plots to LOTs up to the first occurrence of the parity operators. This allows a closer analysis of the occurrences of each operator for the LOTs with the highest correlation with ANN learning effort. The shape of the curves shows where each operator occurs most often. For instance, a straight curve means that the operator appears with a constant frequency, as is the case with negation. On the other hand, a convex curve means that the operator appears more frequently among the LOTs with stronger *ρ*. This is the case for all operators, reflecting the fact that LOTs with more operators in general, rather than with specific operators, tend to have stronger *ρ* (this point is explored more in detail in Figure 4).

The main question is answered in the positive, as displayed in the top plot of Figure 3.^13^ The main result when considering rank correlation across all LOTs is therefore that there is an overall positive rank correlation between logical complexity and ANN learning effort. This means that categories that are simpler in a logical sense can be learned faster by the ANNs.

Figure 3 also informs us as to which LOTs best correlate with the ANN learning efforts. As the top plot shows, depending on the specific LOT, the rank correlation goes from weak (0.22) to strong (0.77). This shows that there is interesting and substantial variation in how well ANN learning effort correlates with different LOTs. We find two main characteristics of LOT that account for the correlation, namely, whether the LOT has parity operators and how many operators are in the LOT.^14^

First, as the bottom plot in Figure 3 shows, much of the variation in rank-correlation is explained by the presence or absence of the parity operators in the LOT. The categories’ logical complexity in LOTs without parity operators rank correlates well with ANNs’ learning effort, with a stark decrease for LOTs with a parity operator. This result is consistent with the previous work discussed above showing that nonlinearly separable categories are particularly hard for ANNs to acquire, since nonlinearly separable categories are those that receive short descriptions in LOTs with parity operators.

**Figure 4.**
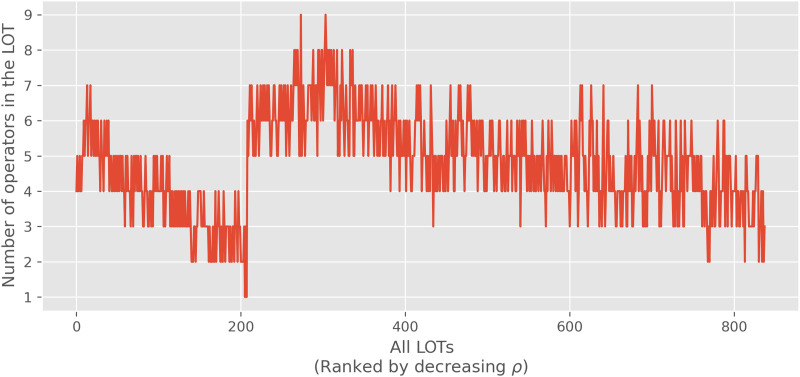
**Number of operators in each language of thought (LOT).** On the *x*-axis, LOTs are ordered in decreasing rank correlation with artificial neural network (ANN) learning effort. For instance, the LOT displayed at *x* = 0 has the highest rank correlation with ANNs, and the rank correlation becomes lower and lower for successive LOTs. The plot shows that both for LOTs with and without the parity operator, having more operators generally increases the rank correlation. As shown in the plot, the maximum number of operators without parity operators (before the transition at ≈ 200) is seven, which increases to nine if the parity operators are included (after the transition at ≈ 200).

The correlation data also shows a second pattern. Among LOTs without a parity operator (and also among the LOTs with a parity operator) there are differences in rank correlation to ANN learning efforts. Namely, LOTs with more operators have higher rank correlations, as can be seen in Figure 4. While the reason for this is not transparent, the explanation might have to do with the fact that LOTs with more operators have more ties in their ranks, since they have a smaller range of minimal formula lengths needed to express all categories. This is a consequence of the fact that the minimal formula length for a category in an LOT is upperbounded by the minimal formula length for that category in any LOT with a subset of its operators.

The interpretation of these results depends on whether one sees connectionism and LOT models as being competing accounts or as belonging to different levels of description (computational for the LOT model, implementational for the ANN model). If so, our article can be seen as a contribution to the project of understanding the relation between different levels of explanation of cognition. In general, accounts of cognition at different levels of analysis can inform each other. Sometimes, they do so by ruling out possible accounts. For instance, considerations of computational complexity can restrict the class of possible algorithms a brain might be implementing (Van Rooij, 2008). In other cases, support is more graded, with one account increasing another account’s plausibility. However, establishing the degree of compatibility between two accounts at different levels of abstraction is not trivial in practice. In the ideal scenario, an explicit characterization is given of the way that higher levels reduce to lower ones. Connectionism and LOT are, however, too complex to directly establish whether the reduction can be done.

Instead, we take the natural approach of comparing the behavioral predictions of the two accounts in the rich domain of category acquisition. If the predictions of the two accounts strongly diverged, this would constitute evidence against the possibility of a reduction. On the other hand, if they are in agreement, this is prima facie evidence for their compatibility, and the two accounts stand or fall together vis-à-vis experimental data from that behavioral domain.^15^ Our article shows that there is considerable agreement between a simple connectionist model and some but not all specifications of the LOT, in terms of their predictions for complexity of category acquisition. We believe therefore that a main contribution of this article is to pave the way to a disentanglement and comparison of the predictions made by connectionism and the LOT hypothesis.

In this light, the two results presented above are significant as they suggest possible avenues for empirical investigation. The first result shows that if independent evidence were to support an LOT model of human cognition, the extent to which such a model would also support a connectionist account would depend on which LOT the data supports. Specifically, our model shows that learning data supporting an LOT model with parity operators would *not* support a connectionist account, while evidence in favor of an LOT without parity operators would mostly also support a connectionist account. The second result, though to a lesser extent, has similar implications for the number of operators in the LOT.

Of course, one could also see the ANN and the LOT models are competing models. In this perspective, our model shows that depending on the operators in the human LOT, learning data will be more or less capable of providing evidence that disambiguates between the two models. If the human LOT has parity operators or contains fewer operators, data from category learning experiments will be more capable of uniquely supporting one of the two models.

## CONCLUSIONS

Much work has been devoted to the problem of Boolean category learning both in the tradition of LOTH and in connectionism. Both of these traditions have pointed to learning effort as a crucial empirical prediction made by the models. However, the relation between the predictions made by the two accounts with respect to learning effort has not been investigated. In the model above, we have presented an initial investigation of this relation. We first formulated predicted learning efforts for the LOTH and for a simple connectionist model in the case of Boolean categories. A comparison between them shows that they are correlated. This indicates that the empirical content of the two accounts is similar for the case of Boolean categorization.

Arguably, one limitation of our results is that we only consider categories definable with up to four properties. By the invariance theorem, the differences in the logical complexity of a given category across LOTs only differ up to an additive constant, which becomes small in the limit of complex enough categories (Li & Vitányi, 1990). A first barrier to increasing the number of properties is computational. Since the complexity of finding the minimal formula increases exponentially with the number of properties, running full simulations with more properties would be impractical. One option would be to only calculate the minimal formulas for some of the categories definable with more operators. However, we believe that beyond the computational limits, the results for few number of properties are in fact the most important in the context of cognitive science. Precisely because of the exponential complexity, we know that either humans are using categories that only depend on a few properties, or they are using heuristics instead of the most compact encoding of the category. Since we do not have any account of what such heuristics could be, the most useful approach at the moment is to consider a set of categories small enough that minimal formulas can be found exactly. This has a better change of being close to the way humans are in fact encoding these categories, should the LOTH be correct.^16^

While we used a general purpose ANN architecture to make our results as general as possible, the results can be extended in various directions. From the connectionist side, our analysis focuses on one specific choice of ANN architecture, model parameters, and training regime. Future work can explore to what extent the results presented above are robust with respect to other choices. A particularly natural extension is not to consider just feedforward ANNs, but rather previous connectionist models of Boolean category acquisition.

Different choices can also be made from the LOT side. In the model we discussed, different LOTs are distinguished by their syntactic primitives. However, Boolean LOTs can also vary with respect to the set of rules that construct formulas from the syntactic primitives. Popular options for the shape of formulas are DNFs (disjunction of conjunctions of literals and their negations), conjunctive normal forms (conjunction of disjunctions of literals and their negations), Blake canonical form, and algebraic normal form. In the model above we did not restrict the shape of the formulas at all, and considered all well-formed formulas in the usual grammar for propositional logic with the LOT’s operators. However, such restrictions might make a difference. Consider again the example of the Boolean LOT with the two properties pictured in Figure 1 above and the operators ¬, ∧, and ∨. However, assume now that every formula is in DNF. Category 13 can be then encoded as (*p* ∧ *q*) ∨ (*p* ∧ ¬*q*) ∨ (¬*p* ∧ *q*). This example shows that restricting sentence form, for example, to DNF, can lengthen the shortest formula: without this restriction category 13 could be expressed simply as *p* ∨ *q*. Previous work has looked at some ways to constrain the shape of formulas in the context of Boolean LOTs (Piantadosi et al., 2016). However, systematically exploring the space of Boolean grammars is a much harder task than exploring the set of primitives, and therefore we leave the task to future work.

A limitation of the article is its focus on the Boolean domain. There is extensive literature showing how LOT models, enriched with probabilistic tools, show learning behavior resembling that of humans. For instance, this has been shown in domains such as taxonomies (Tenenbaum et al., 2006), handwritten symbols (Lake et al., 2015), kinship terms (Mollica & Piantadosi, 2022), and numerical concepts (Piantadosi et al., 2012). Of course, analyzing these domains would go beyond the scope of this article. Nonetheless, they suggest further ways of comparing learning patterns of ANNs and LOT models.

Connections to other previous work can also be analyzed more in depth. One particularly relevant paper is Griffiths et al. (2012), which compares the inductive biases of ANNs and a Bayesian learner. Our article differs from Griffiths et al. (2012) in at least three important ways. First, Griffiths et al. (2012) uses linear activations functions, restricting the expressive power of the ANNs compared to the ones usually used in the literature. Second, they do not use a generatively structured hypothesis space, but rather directly infer a matrix. Third, they only consider a restricted set of possible bases for the Bayesian inference, rather than systematically exploring a set of possible LOTs.

Despite its limitations, this article has shown that connectionism and many LOTs have similar empirical import in the case of learning effort for Boolean categorization. One suggestive explanation for this is that they both approximately track the underlying complexity of the functions characterizing each category. While complexity cannot be computed exactly, there are methods to approximate it. Showing a correlation between the measures presented in this article and approximated complexity would therefore provide evidence for a unified account based on complexity. We leave these possible developments to future work.

## ACKNOWLEDGMENTS

The research leading to these results has received funding from the European Research Council under the European Union’s Seventh Framework Programme (FP/2007-2013) / ERC Grant Agreement n. STG 716230 CoSaQ. We thank Steven Piantadosi and Shane Steinert Threlkeld for the interesting discussion that was inspiring for this project.

## FUNDING INFORMATION

JS, Seventh Framework Programme (https://dx.doi.org/10.13039/100011102), Award ID: STG716230.

## AUTHOR CONTRIBUTIONS

FC: Conceptualization: Equal; Formal analysis: Lead; Methodology: Lead; Visualization: Lead; Writing - original draft: Lead; Writing - review & editing: Equal. JS: Conceptualization: Equal; Formal analysis: Equal; Visualization: Equal; Writing - original draft: Equal; Writing - review & editing: Equal.

## Notes

^1^ See, for example, Davies (1991) and Fodor and Pylyshyn (1988) for classical discussions and Rescorla (2019) for an overview of the debate. See Gallistel and King (2009) for discussion of empirical case studies in support of LOTH and against connectionism.^2^ Though see Vigo (2006) for a discussion of a technical problem in the original paper.^3^ Classical studies in a similar paradigm include Shepard et al. (1961), which studies the generalization errors of different Boolean categories, and Bruner et al. (2017) in the constructivist program. In contrast to more recent work, this work does not explicitly model the learning process in terms of an LOT, making a direct comparison difficult.^4^ Alternatively, we can think of an object as an equivalence class of entities that are indistinguishable with respect to the chosen properties. If we consider all properties humans can represent, by definition entities within these equivalence classes must be treated equivalently by the LOT.^5^ Note that there is a correspondence between the set of categories and the set of functions from objects to a Boolean.^6^ To be more precise, if “LOT” is taken to refer to the whole language underlying human thinking, it is more appropriate here to talk of the Boolean *fragment* of the LOT. In the following we just talk of “Boolean LOT” for simplicity.^7^ A large literature in computer science concerns itself with the similar problem of finding the smallest circuit for computing Boolean functions, for example, Wegener (1987). This literature is mostly focused on finding efficient algorithms for minimization with specific operators or showing general results on bounds of circuit complexity. Therefore, it will mostly not be relevant for the present purposes.^8^ Such sets of operators can also be described as *functionally complete*. The sets of operators usually considered are bases. To get a sense of a functionally incomplete sets, consider the LOT {p, q, or}. With such an LOT, it is not possible to define the category consisting of only the object that is neither p nor q. To get the set of bases, we calculated the powerset of the set of nine operators described above, and then filtered the supersets of the known list of minimal bases, as described, for example, in Wernick (1942).^9^ This is often also called XOR (exclusive OR) in the literature.^10^ Moreover, Mhaskar et al. (2016) shows that depth also has an impact on how efficiently the functions can be represented. See Anthony (2010) for a more detailed discussion of the representation power of various types of ANN.^11^ See Penny and Stonham (1992) for a discussion of Boolean function learning with *probabilistic logic nodes* (PLN), which can be implemented directly in RAM. Moreover, see Deolalikar (2001) for an analysis of Boolean category acquisition in ANNs comprising only neurons with zero thresholds and binary weights.^12^ See Mazzoni and Wagstaff (2004), Pérez and Rendell (1995), and Settles and Craven (2008) for other examples of area under the curve being used as measure of learning. See Settles and Craven (2008) for a more general discussion of learning curves of ANNs.^13^ Readers interested in replicating the results can find the needed code at https://github.com/thelogicalgrammar/ANN_complexity. The data reported in the article can be found at https://osf.io/gfsdq/.^14^ Interested readers can find a plot of the by-category average minimal formula length vs. ANN learning effort at https://thelogicalgrammar.github.io/ANN_complexity/hover_cat.^15^ Moreover, if their predictions agree more than any other combination of plausible accounts, this lends evidence to the idea that the higher level one indeed reduces to the lower level one. In our case, we have not compared our computational-level account (the LOT) with other alternatives, and therefore cannot yet assess this situation.^16^ For a thorough discussion of the role that considerations of computational complexity can play in theory building in the cognitive sciences, see Van Rooij (2008).

## APPENDIX: INTERPRETING ANN INPUT/OUTPUT

The situation is complicated somewhat by the fact that each input/output relation can be interpreted by the artificial neural network (ANN) as different categories depending on whether 0 and 1 are interpreted as meaning *True* or *False* (the interpretation of binary values is given independently for input and output). For instance, consider the following possible input/output relation for an ANN (with only two objects instead of four for ease of visualization):

$$\left\{\begin{array}{l} \left(0,0\right)\Rightarrow 1\\ \left(0,1\right)\Rightarrow 0\\ \left(1,0\right)\Rightarrow 0\\ \left(1,1\right)\Rightarrow 0 \end{array}\right\}$$

This can be interpreted as encoding different categories as follows (where each cell gives the category as a set of sets of properties):

**Table:**

	1 in input as True	1 in input as False
1 in output as True	{¬*p*, ¬*q*}	{*p*, *q*}
1 in output as False	{¬*p*, *q*}, {*p*, ¬*q*}, {*p*, *q*}	{*p*, ¬*q*}, {¬*p*, *q*}, {¬*p*, ¬*q*}

From a total of 65,536 possible categories (with four objects), we train the ANNs on 32,768 input/output relations, and interpret the learning efforts as representative of both interpretations of the input binary values.

## Supplemental Materials

https://github.com/thelogicalgrammar/ANN

https://osf.io/gfsdq/
